# Costs of Foraging Predispose Animals to Obesity-Related Mortality when Food Is Constantly Abundant

**DOI:** 10.1371/journal.pone.0141811

**Published:** 2015-11-06

**Authors:** John M. McNamara, Alasdair I. Houston, Andrew D. Higginson

**Affiliations:** 1 School of Mathematics, University of Bristol, University Walk, Bristol, BS8 1TW, United Kingdom; 2 School of Biological Sciences, Life Sciences Building, University of Bristol, 24 Tyndall Avenue, Bristol, BS8 1TQ, United Kingdom; 3 College of Life and Environmental Sciences, University of Exeter, Exeter, EX4 4QG, United Kingdom; University of St Andrews, UNITED KINGDOM

## Abstract

Obesity is an important medical problem affecting humans and animals in the developed world, but the evolutionary origins of the behaviours that cause obesity are poorly understood. The potential role of occasional gluts of food in determining fat-storage strategies for avoiding mortality have been overlooked, even though animals experienced such conditions in the recent evolutionary past and may follow the same strategies in the modern environment. Humans, domestic, and captive animals in the developed world are exposed to a surplus of calorie-rich food, conditions characterised as ‘constant-glut’. Here, we use a mathematical model to demonstrate that obesity-related mortality from poor health in a constant-glut environment should equal the average mortality rate in the ‘pre-modern’ environment when predation risk was more closely linked with foraging. It should therefore not be surprising that animals exposed to abundant food often over-eat to the point of ill-health. Our work suggests that individuals tend to defend a given excessive level of reserves because this level was adaptive when gluts were short-lived. The model predicts that mortality rate in constant-glut conditions can increase as the assumed health cost of being overweight decreases, meaning that any adaptation that reduced such health costs would have counter-intuitively led to an increase in mortality in the modern environment. Taken together, these results imply that efforts to reduce the incidence of obesity that are focussed on altering individual behaviour are likely to be ineffective because modern, constant-glut conditions trigger previously adaptive behavioural responses.

## Introduction

Obesity is a major and growing medical and social problem in humans, domestic and captive animals [1,2] because excessive fat storage results in ill health and mortality through several physiological impacts on the functioning of the body [3–7]. Interventions to help people to lose weight have limited success in that body weight tends to be resistant to change [8–10], for reasons that are not well understood [11]. Progress in understanding fat storage has frequently followed from research on the adaptive use of energy reserves by animals [12–17]. Whilst the various health risks suggest obesity is not adaptive, maladaptive behaviours can emerge from behavioural strategies that are adaptive in natural environments [18,19]. Thus, it is likely to be beneficial to understand how selection in natural environments has resulted in feeding strategies that now promote persistent obesity [17,20–22]. That is, we need to identify the environmental challenges that have led to a phenotype that maintains an unhealthy level of energy reserves.

Typically, researchers have assumed that the important environmental challenge has been the occasional instances of food shortage [23]. The thrifty genotype hypothesis for obesity supposes that the storage of fat prepares an individual for a famine that, in the modern environment, never arrives [20,24,25]. The risk of starvation [25], catabolism of important proteins [26] and reduced immune function [17] when underweight are proposed to have selected for weight gain when food is available. Following this strategy in the modern Western environment, which has been likened to a ‘continuous feast’ [27,28], can lead to overweight and obesity. Even so, in the pre-modern environment it is unlikely that food shortages are sufficiently common to prevent animals becoming overweight, nor is it the case that food availability is only barely sufficient to maintain energy balance the rest of the time. Furthermore, it is not clear why animals have evolved the desire to store excess fat but not to avoid morbidity costs of being overweight or obese, because if animals will gain a large amount of weight when it is possible, we would expect that natural selection would have reduced or removed the health costs of being overweight.

The dynamics of the food supply in natural environments are not limited to ‘barely sufficient’ and ‘insufficient’: often there will be gluts of food, such as during brief wet seasons, whilst trees are fruiting, and at harvest time [17]. Such gluts may provide a reason for individuals to consume more food than they should maintain in the long term. Gluts are likely to be short-lived and so fat will eventually be used up, but during periods when fat stores are above the long-term requirements, the animal does not have to look for food to meet its needs. A major cost of foraging in animals is exposure to predators [29–32] so the risk of predation is a reason to avoid foraging when possible. Furthermore, when not foraging animals are typically carrying out other behaviours that increase reproductive success. For instance, many mammals gain weight when food is abundant and eat little during the breeding season [33]. The need to forage can therefore be considered to be a cost that prevents investment in reproduction. In such conditions, the selective pressure for changes in physiology that reduce the long-term costs of being overweight will typically be weak because the extra reserves are not maintained long-term. However, the ‘constant glut’ conditions in artificial environments have led to high levels of obesity [27] and the health costs are significant. In this paper we show that by considering the optimal behaviour in response to both gluts of food and the costs of foraging–two key features of natural environments–we are able to understand current behaviour and make predictions about current levels of mortality from obesity. The starvation-predation trade-off has been successful at predicting levels of body fat across animal species [34]. Here, we show that this trade-off could have interacted with the occurrence of gluts of food to select for strategies that cause maladaptive fat storage in artificial environments.

## The Model

We consider an animal that is attempting to survive a long period in which it does not reproduce in an environment with rare and short-lived gluts of food. The animal faces three sources of mortality: death due to low fat reserves (e.g. starvation due to food shortage, or disease due to a poor immune system), obesity-associated morbidity, and predation while foraging. For clarity we assume that the mortality from predation when resting is zero, but our argument only depends on external mortality (from predation, injury from defensive prey, accident, conflict with other humans, or disease) being greater when foraging than when resting. The mortality rates from these three sources depend on how much fat the animal stores. The rate of starvation is assumed to decrease with the level of fat maintained because the animals can survive a longer period without food or invest more in the immune system. In this general approach we do not explicitly model the actual mechanism, but the assumed rate of mortality represents them. The rate of predation while foraging is assumed to increase with the current level of fat due to increased vulnerability because of reduced mobility [35–37]. The rate of obesity-related mortality is assumed to increase with reserves at an accelerating rate, as observed in studies of humans [38].

We consider that natural selection on the animal (e.g. Palaeolithic hunter-gathering humans) has led to a fat-storage strategy that minimises the total rate of mortality [29,39]. A strategy is specified by two target levels of fat: the amount of fat to store during normal conditions (*F*_*N*_) and the level of fat to build up to during a glut (*F*_*G*_). Consider the animal under normal conditions. Suppose that the animal alternates between foraging and resting so as to maintain its fat reserves at level *x*. The animal will be subject to three sources of mortality; predation risk while foraging, the risk of starvation should a food shortage occur and mortality due to the level of fat carried. Let *A*(*x*) denote the average mortality rate of this animal. Then over a time interval of length *T* the probability that the animal does not die is

$$S_{T}\;=\;\text{exp}\left\{-\int_{0}^{T}A(x)dt\right\}\;=\;e^{-A(x)T}.$$

Thus for any *T*, the probability it survives the time interval is maximized by choosing *x* to minimize *A*(*x*). We denote the level of fat reserves *x* at which this minimum is achieved by $F_{N}^{*}$.

**Fig 1:**
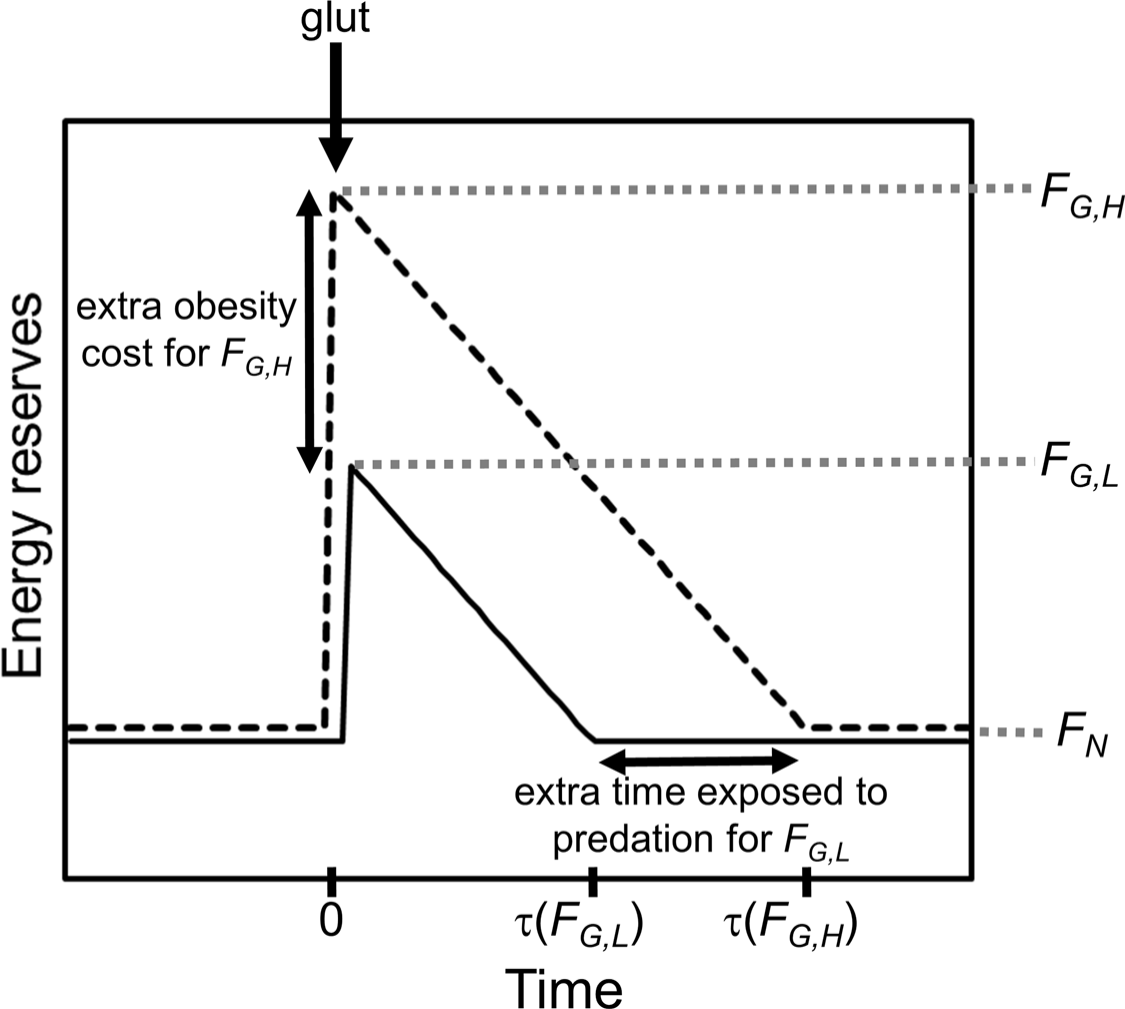
Illustration of the trade-off between the mortality rates from obesity and predation when setting the target level of reserves during a glut (*F*_*G*_). The two illustrated strategies have the same target under normal conditions (*F*_*N*_) but different *F*_*G*_ values. The higher *F*_*G*_ means that when a glut occurs reserves increase by a larger amount, which enables the animal to avoid foraging for a long time before reserves decrease to *F*_*N*_. However, at such high reserves the mortality rate from obesity is higher. The lower *F*_*G*_ avoids the period of very high reserves (labelled ‘extra obesity cost’) but reaches *F*_*N*_ sooner, and so incurs extra mortality from predation (‘extra time exposed to predation’).

Now suppose that the animal is maintaining reserves at $F_{N}^{*}$ when a rare glut of food occurs. We assume that during a glut the animal can gain as much fat as it likes very quickly and that during this time the rate of predation is negligible compared to baseline. By the end of this time, conditions have returned to normal. Building up fat during a glut enables the animal then to forgo foraging for the time it takes for fat levels to fall from *F*_*G*_ to *F*_*N*_, and so avoid the risk of predation during that time. However, the mortality rate associated with obesity increases with *F*_*G*_, which limits the benefit of fat and leads to a trade-off between obesity-related mortality and mortality from predation (Fig 1).

### Mortality rates under the optimal strategy

Here, we show that the animal should build up reserves during a glut to the level where the total mortality when resting is equal to the total mortality under normal conditions. We denote the critical levels of *F*_*N*_ and *F*_*G*_ that minimise the total mortality rate by $F_{N}^{*}$ and $F_{G}^{*}$ respectively. Since gluts are rare, $F_{N}^{*}$ is not influenced by what happens during gluts and just minimises the total mortality rate under normal conditions. Suppose that during the glut the animal builds up its fat reserves to level *y* and this build up is essentially instantaneous and is achieved at no additional risk of predation. The animal then rests until fat reserves fall to $F_{N}^{*}$ again. Suppose that this takes time *τ*(*y*). Once fat reserves fall to $F_{N}^{*}$ the animal maintains its reserves at this level. Then the probability the animal is still alive time *T* after the glut (where *T* > *τ*(*y*)) is

$${\hat{S}}_{T}(y)\;=\;\text{exp}\left\{-(\int_{0}^{\tau (y)}R(x(t))dt\;+\;\int_{\tau (y)}^{T}A(F_{N}^{*})dt)\right\},$$

where *R*(*x*) is the mortality rate while resting at reserve level *x* and *x*(*t*) is the animal’s fat reserves at time *t*. We rewrite this survival probability as

$${\hat{S}}_{T}(y)\;=\;\text{exp}\left\{-(\int_{0}^{\tau (y)}[R(x(t))-A(F_{N}^{*})]dt\;+\;\int_{0}^{T}A(F_{N}^{*})dt)\right\}.$$

Since the second integral does not depend on *y*, survival is maximized (for all *T* > *τ*(*y*)) by minimizing

$$I(y)\;=\;\int_{0}^{\tau (y)}[R(x(t))-A(F_{N}^{*})]dt.$$

Changing the integral to one over *x* rather than *t* (using the fact that *x*(0) = *y* and $x(\tau (y))=F_{N}^{*}$) gives

$$I(y)\;=\;\int_{y}^{F_{N}*}\left[R(x)-A(F_{N}^{*}\right)]\frac{dt}{dx}dx$$

Differentiating with respect to *y* we obtain

$$I\prime (y)\;=\;[R(y)-A(F_{N}^{*})](-\frac{dt}{dx}(y)).$$ (1)

Note that, since reserves decrease during resting we have $-\frac{dt}{dx}(x)>0$ for all reserves *x*.

Since resting incurs a lower mortality rate than active foraging we have $R(F_{N}^{*})<A(F_{N}^{*})$, so that *I*′(*F*_N_*) < 0. We assume that the mortality increase with fat reserves is such that $R(y)>A(F_{N}^{*})$ for all sufficiently large *y*, so that *I*′(*y*) > 0 for all sufficiently large *y*. Then *I*(*y*) has a minimum value at some value $y=F_{G}^{*}$ that satisfies $I\prime (F_{G}^{*})=0$. By Eq (1) it follows that

$$R(F_{G}^{*})=A(F_{N}^{*}).$$

That is, the optimal *F*_*G*_ is the level of reserves at which the total mortality when not foraging (i.e. due to starvation and obesity, but not predation) is equal to the overall mortality rate under normal conditions when maintaining fat levels at $F_{N}^{*}$. To understand this result, recall that building up fat during a glut allows the animal to rest safe from predators for a while. The more fat that is put on, the longer the period of rest. Assuming that at the start of the period of rest the fat level is higher than under normal conditions and the mortality rate while resting is less than the normal mortality rate, it is worth putting on more fat to prolong the period of rest still further.

### Implications for obesity

In many modern human societies and the artificial environments in which domestic animals are kept, food is always abundant (i.e. glut is the usual condition). If there has been insufficient time for natural selection to act on the pre-modern adaptations then animals still follow the rule that is appropriate for the natural environment, in which gluts are rare, and their level of fat will always be $F_{G}^{*}$. It follows from our result that the mortality rate (from starvation and obesity-related illness) in the current predator-free environment should equal the average mortality rate in the environment in which animals (including humans) evolved. Since the rate of starvation is likely to be small when the individual has large fat stores, we conclude that the obesity-related mortality rate in constant glut conditions should approximately equal the average total mortality rate in the pre-modern environment.

**Fig 2:**
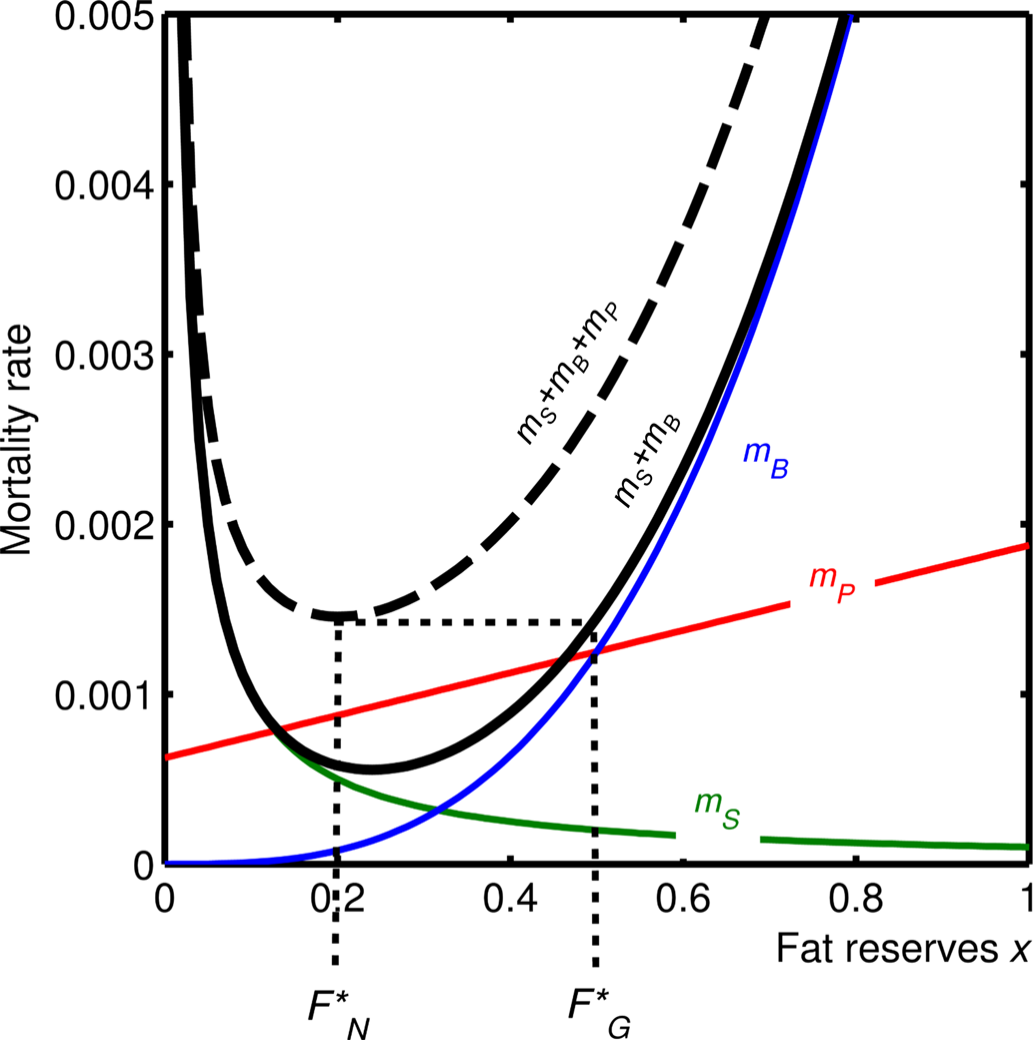
Illustration of the mortality rates *m*_*i*_ as a function of reserves *x* when foraging at the appropriate rate to maintain reserves at *x* (dashed line) and when resting (solid line) for a specific model implementation (see Appendix). The mortality from starvation (*m*_*S*_, green line) declines rapidly with increasing fat reserves *x*, and so is only substantial at low reserves. The mortality from obesity (*m*_*B*_, blue line) is small at low reserves and increases at an accelerating rate. Mortality due to predation during foraging (*m*_*P*_, red line) increases with reserves. The optimal normal fat level $F_{N}^{*}$ is at the minimum of the sum of the mortality rates under normal conditions (*m*_*S*_ + *m*_*B*_ + *αm*_*P*_). During a glut, the animal should feed up to reserves level $F_{G}^{*}$ where the mortality from obesity causes total mortality (not including *m*_*P*_, which is zero while resting) to equal that at $F_{N}^{*}$ (dotted lines). It can then rest, avoiding the risk of predation, until its reserves fall to $F_{N}^{*}$, whereupon it must start to forage again before it suffers a large increase in mortality due to low reserves. Parameter values: γ = 10, ψ = 3, μ = 0.005, ϕ = 2, β = 3, κ = 0.01, ρ = 0.0001.

Fig 2 illustrates this result for some example forms and parameters of the mortality functions (see Appendix). For these functions, most mortality during normal conditions is due to predation, in line with many observations of wild animals (e.g. [40,41–44]), and obesity-related mortality is rare but present, consistent with the observation that non-obese people do, but less commonly, suffer from obesity-related diseases [3–6]. As a consequence, our result means that current obesity-related mortality is approximately equal to mortality *from predation* in the natural environment.

**Fig 3:**
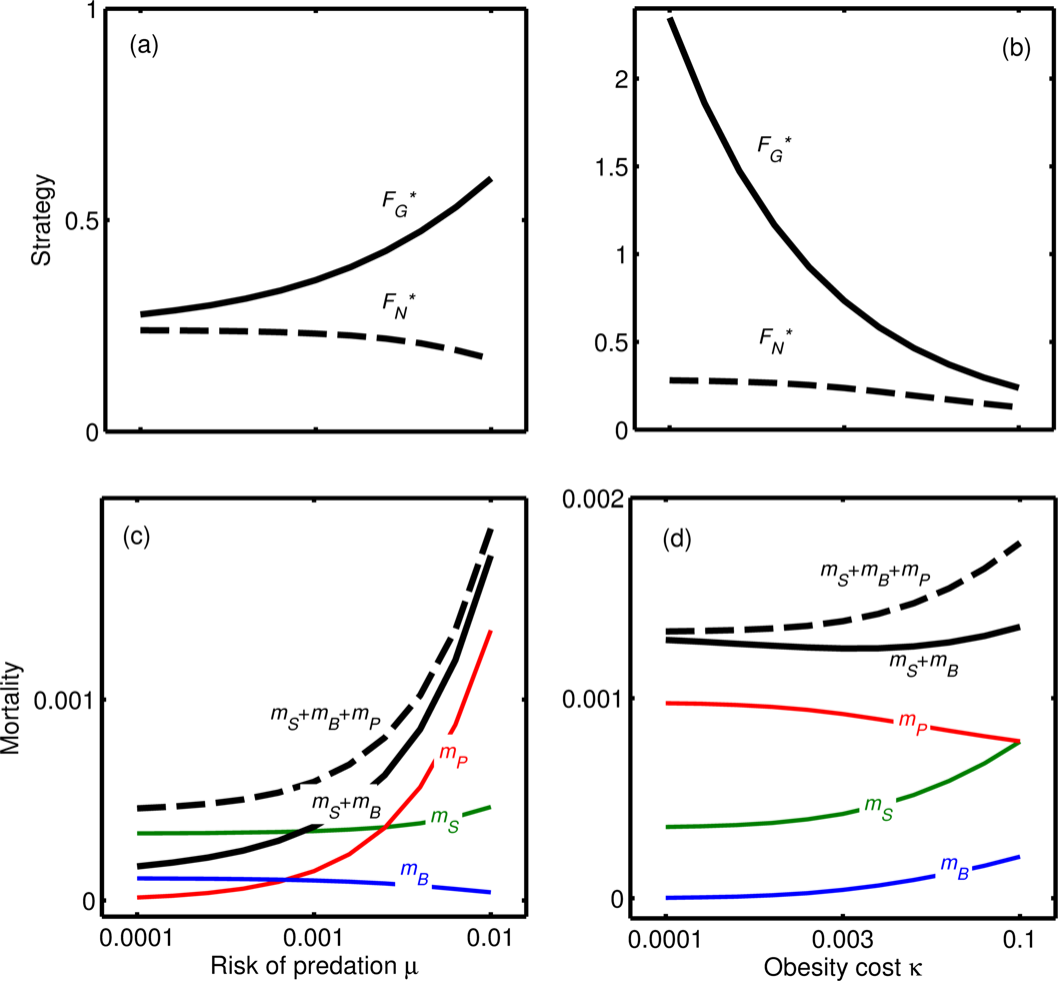
Effects of varying (a, c) risk of predation μ and (b, d) the cost of obesity κ on (a, b) the optimal strategy (dashed lines: $F_{N}^{*}$, solid lines: $F_{G}^{*}$) and (c, d) the various sources of mortality under normal conditions (starvation *m*_*S*_, green lines; predation *m*_*P*_, red lines; obesity *m*_*B*_, blue lines), total mortality under normal conditions (dashed lines), and mortality from obesity in constant glut (solid lines). Note that the mortality from obesity under normal conditions is always very small and that at low κ mortality under constant glut conditions *increases* (to a small extent) as κ decreases. Baseline parameter values: γ = 10, ψ = 3, μ = 0.005, ϕ = 2, β = 3, κ = 0.01, ρ = 0.0001.

As predation risk increases, animals should be less fat in normal conditions to reduce the risk of predation, and feed more in gluts to enable a longer resting period (Fig 3A). Mortality in gluts will therefore increase with natural predation risk (Fig 3C). As the obesity cost (*κ*) decreases, weight gain during gluts increases (Fig 3B), mortality in normal conditions decreases whilst mortality in constant glut conditions may even increase (Fig 3D). That is, lowering the cost of being overweight may not reduce the predicted obesity-dependent mortality rate during a constant glut, and may even increase it, since a lower cost results in greater fat storage in a glut (i.e. larger $F_{G}^{*}$). We can now answer the following question: If carrying high fat loads had a health cost why hasn’t natural selection acted on our physiology to reduce this cost? In the context of our model, the deleterious effects of high fat levels would mostly operate in a short period after rare gluts. This suggests that the selection pressure on animals to change their physiology has been low. Furthermore, any selection that did act to reduce the intrinsic health costs of obesity could have counter-intuitively led to *increased* obesity (and obesity-related mortality) in constant-glut conditions.

## Discussion

Our simple evolutionary model provides a novel hypothesis for the functional causes of obesity in domestic animals (e.g. humans, pets and laboratory subjects) in altered environments to which they are not yet adapted, where food is abundant and foraging carries no predation risk. Overweight individuals do not gain weight indefinitely, but maintain a given, high level of fat reserves [10,45]. Any explanation for obesity must provide reasons why selection has not led to a weight control system that avoids storing a maladaptive level of fat, why the system appears to defend a level of fat that is too high, and how being overweight can still be unhealthy if adaptive fat storage strategies cause it to occur. Our model suggests that these are all consequences of an artificial environment that triggers an adaptive response. That is, selective pressures from risky foraging and a variable food supply have favoured a feeding strategy that attempts to build up fat reserves to a deleterious, fixed, level when food is abundant.

We have explored what the evolutionary consequences would be in the assumed conditions. We now assess the extent to which our model applies to humans. Existing approaches to understanding obesity focus on the usefulness of fat as an insurance against a shortage of food. However, there is much doubt about whether food shortages were sufficiently common to exert a strong selective pressure on ancestral human populations [46]. The thrifty genotype and thrifty phenotype hypotheses are based on the idea that the body prepares for periods of food shortage [20]. However, it is unlikely that food shortages were sufficiently common nor food under normal conditions sufficiently restricted to prevent humans in ancestral environments becoming overweight [47]. It is therefore necessary to explain why humans are not overweight in natural conditions but become so in constant-glut conditions, and do so even if the starvation rate has always been low. Our explanation follows from the acknowledgment that storing energy is also a way to avoid the need for foraging and its associated costs (e.g. predation) for some time. In many traditional societies people experience occasional gluts of food and practice ritual feeding [48,49], which allows them to avoid intensive foraging when food is scarce. The innovations of weaponry and the control of fire did not eliminate the risk of mortality from predation and other sources while foraging for humans [50,51], so selection for avoiding predation will have influenced the evolution of current human feeding behaviour. Now, of course, there is essentially no predation risk, which may lead eventually to a lower target as humans evolve.

In line with work on the use of fat by animals, we assume that the selective pressure against foraging is from predation [31,34] and that the risk of predation is assumed to increase with fat stores. This is based on observations of reduced mobility demonstrated in birds [35–37], so it would be useful to have studies of mobility and predator evasion in running animals. We have also assumed that mortality from obesity is instantaneous in the same way as predation, when in reality morbidity occurs due to chronic overweight or obesity [4,5,52]. Further work with a completely new model is needed to investigate how model predictions are changed when the effects of obesity are cumulative. Nevertheless, we would expect the insights gained from our current model to still apply. Our model does predict that some individuals will suffer from obesity-related diseases even when not obese (i.e. *m*_*B*_>0 even when *F*_*G*_ is not large in Fig 3), and these may be the so-called metabolically unhealthy lean people. However, these values are quite small, so mortality from obesity-related diseases is predicted to be high only when individuals are obese, as is observed in humans [3–7]. One further issue with our hypothesis is the challenge to explain how selection could act on body weights that were unachievable in pre-modern environments (hunter-gatherers typically do not become obese [53]).

All of these issues can be resolved by considering that our model is a conceptual one that illustrates the idea of a response to glut that reduces the need to forage; the real situation need not match exactly how we have discussed it. We have described the three costs in our model as mortality rates, but the hypothesis also works if the three costs are ongoing impacts on reproductive value: starvation could be substituted by any cost of having low reserves that reduces breeding ability, such as a compromised immune function [4]; obesity-related morbidity need not be fatal to affect reproductive success [54,55]; and the increasing costs of foraging may not be predation rate but that time spent not foraging is invested in other activities that increase reproductive fitness, meaning it would then be worth gaining a great deal of weight during glut and relying on this energy whilst, for example, competing for mates. We have assumed that gluts are rare and instantaneous, reflecting the idea that an abundance of fruit or animal prey would be short-lived compared to the duration of normal conditions, the length of food shortages, and the impacts of obesity on health. Our predictions would not be qualitatively affected if we assumed that feeding up in gluts took time and predation occurred during gluts provided that the predation risk associated with foraging is lower than that under normal conditions.

Our results on the role of predation (Fig 3A and 3C) allow us to speculate about a possible reason for between-individual variability in the amount of energy reserves that individuals store. Variability in the amount of fat stored by individuals may be due to variability in the subconscious perception of risk, which may explain observed correlations between obesity and anxiety [2,56]. Furthermore, under this view we would not expect any individual gene to have strong effects on body weight, since the tendency to eat will be subject to a range of genetic and environment influences that all affect responses to risk and rewards. Our perspective enables us to provide an explanation rooted in evolutionary theory for why not all individuals become obese and why even populations in the most affluent societies maintain a very wide range of body weights.

Current efforts to reduce the incidence of obesity are largely focussed on influencing the behaviour of individuals that are viewed as having a disease. Our results suggest that a different perspective might lead to more successful medical interventions. In our model, overweight people are viewed as simply following feeding strategies that were adaptive in ancestral humans but not in modern, constant-glut conditions. Such a fundamental innate strategy for ensuring survival is likely to be resistant to conscious control, and so it should be unsurprising that treatments for obesity are ineffective [2]. Whilst we would expect humans to adapt to the constant-glut conditions, this will only occur over evolutionary time, taking many generations. Efforts to reduce the incidence of obesity that are focussed on altering individual behaviour will be ineffective whilst the altered food environment triggers previously adaptive behavioural responses. Future research effort should therefore be focussed on understanding exactly what aspects of the modern environment trigger these naturally selected behavioural responses.

## Appendix: Specific Model Implementation

Here, we provide a full description of a specific implementation of the model and the derivation of results. We assume that the magnitudes of the rates of mortality from starvation (*m*_*S*_), predation (*m*_*P*_), and obesity (*m*_*B*_) are controlled by the scalars *ρ*, *μ*, and *κ* respectively.

We assume that under normal conditions the animal must set its foraging rate to maintain energy balance. At foraging rate *α* and cost of activity relative to resting *ψ*, the energy usage rate is *αψ* + (1 − *α*). The availability of food is *γ* and so the energy gain rate is *γα*, and the animal maintains a constant level of energy reserves, so rearrangement of the energy balance equation gives the foraging rate

$$\alpha =\frac{1}{\gamma -\psi +1}.$$ (A1)

The predation rate is

$$m_{P}=\alpha \mu (1+\phi x)$$ (A2)

where *μ* is predator density and *ϕ* controls the extent to which energy reserves make the animal more vulnerable to predation.

Substituting Eq (A1) into Eq (A2) gives the mortality rate due to predation when the animal balances energy in normal conditions:

$$m_{P}=\frac{\mu (1+\phi x)}{\gamma -\psi +1}.$$ (A3)

The rate of mortality from obesity does not depend on activity level but is mass-dependent, increasing with fat reserves according to

$$m_{B}=\kappa x^{\beta },$$ (A4)

where κ and *β* are fixed parameters. We assume *β*>1 to reflect the trend for the relationship between body weight and the health costs of overweight are accelerating for overweight and obese people [57].

The rate of starvation depends on how often periods of no food availability occur (ρ). For tractability we assume that the distribution of famine durations is such that mortality during famine is described by the reciprocal of reserves (e.g. because most famines are fairly short):

$$m_{S}=\frac{\rho }{x}.$$ (A5)

The total mortality rate under normal conditions is the sum *A* = *m*_*P*_ *+ m*_*B*_ *+ m*_*S*_, so from equations (A3–A5)

$$A(x)=\frac{\mu (1+\phi x)}{\gamma -\psi +1}+\kappa x^{\beta }+\frac{\rho }{x}.$$

Since gluts are rare, the optimal level of reserves under normal conditions *F*_*N*_ * is that which minimises the *A*(*x*):

$$A(F^{*}{}_{N})=\frac{\mu (1+\phi F^{*}{}_{N})}{\gamma -\psi +1}+\kappa F_{N}^{*}{}^{\beta }+\frac{\rho }{F_{N}^{*}}.$$ (A6)

When resting and safe from predators, the mortality rate is

$$R(x)=\kappa x^{\beta }+\frac{\rho }{x}.$$ (A7)

We are interested in the optimal reserves to feed up to in a glut, $F_{G}^{*}$. We show in the main text that $R(F_{G}^{*})=A(F_{N}^{*})$, so we can use Eqs (A6) and (A7) to find $F_{G}^{*}$. Given $F_{G}^{*}$, we can assess mortality from obesity in an obesogenic environment of constant glut and no predation or starvation. Progress is possible only if we assign values to the parameters. The values we used are given in the figure legends.
